# Determinants of Left Atrial Appendage Volume in Stroke Patients without Chronic Atrial Fibrillation

**DOI:** 10.1371/journal.pone.0090903

**Published:** 2014-03-04

**Authors:** Mikko Taina, Petri Sipola, Antti Muuronen, Marja Hedman, Pirjo Mustonen, Anne-Mari Kantanen, Pekka Jäkälä, Ritva Vanninen

**Affiliations:** 1 Department of Clinical Radiology, Kuopio University Hospital, Kuopio, Finland; 2 Unit of Neurology, Institute of Clinical Medicine, University of Eastern Finland, Kuopio, Finland; 3 Unit of Radiology, Institute of Clinical Medicine, University of Eastern Finland, Kuopio, Finland; 4 Heart Center, Kuopio University Hospital, Kuopio, Finland; 5 Department of Cardiology, Keski-Suomi Central Hospital, Jyväskylä, Finland; 6 NeuroCenter, Kuopio University Hospital, Kuopio, Finland; Cardiff University, United Kingdom

## Abstract

**Background:**

Left atrial appendage (LAA) volume has been shown to be increased in patients with acute cryptogenic stroke. Atrial fibrillation (AF) is a well-recognized risk factor but it is not the only one associated with LAA enlargement. The aim of the study was to clarify the multifactorial etiology of LAA enlargement in cardiogenic stroke/TIA patients without AF.

**Methods:**

Altogether 149 patients with suspected cardioembolic stroke/TIA (47 females; mean age 61 years) underwent cardiac CT. Diagnosed AF on admittance was an exclusion criteria but 24-hour Holter ambulatory ECG revealed paroxysmal AF (PAF) in 20 patients. Body surface area adjusted LAA volume was evaluated. Eighteen different variables were registered including general characteristics, definite and potential causal risk factors for ischemic stroke/TIA, clinical echoparameters and CT based cardiac volumetric and adipose tissue measurements. A stepwise linear regression analysis was performed to achieve a model adjusted for the number of predictors of LAA volume increase.

**Results:**

In linear regression analysis, the best model accounted for 30% of the variability in LAA volume, including PAF (19%) and enlarged left atrial volume (6%), enlarged left ventricle end-systolic diameter (3%) and decreased pericardial adipose tissue (2%). No multi-colinearity between variables was observed. In addition to PAF, no other definitive or potential causal risk factors could account for the LAA volume in these patients.

**Conclusions:**

LAA volume increase seems to be poorly associated with currently known stroke/TIA risk factors, except for AF. Targeting more comprehensive ECG monitoring for stroke patients with increased LAA volume should be considered.

## Introduction

Despite improvements in stroke mortality in Western societies, stroke remains the second leading cause of death worldwide [1]. An embolus in the brain provides a well-recognized etiology for ischemic stroke together with decreased perfusion and thrombosis [2]. It has been suggested that cardioembolism may constitute a major mechanism for not only cardiogenic but also cryptogenic stroke [3], [4], [5]. When derived from the heart, an embolus may be induced by either chronic atrial fibrillation (AF) or paroxysmal atrial fibrillation (PAF) via predisposing clot formation and by reshaping of the left ventricle (LV), left atrium (LA), and left atrial appendage (LAA) [6], [7], [8]. Over 90% of all cardioemboli are formed in the LAA [8]. The LAA has been shown to be frequently enlarged in patients with acute cryptogenic stroke or a transient ischemia attack (TIA) [5]. Both AF and PAF are known to contribute to LAA enlargement [9], [10]. However, the factors associated with LAA volume increase are not fully understood.

For the risk stratification of stroke recurrence and accurate anticoagulation, or other treatment targeting, we need a more comprehensive understanding of stroke pathogenesis. In the current study we measured LAA volumes with cardiac CT (cCT) and adjusted the volumes for patient’s body surface area (BSA) [11]. Our aim was to identify the clinical factors and/or imaging findings that are associated with LAA volume increase.

## Methods

The EMBODETECT study and all participants were approved by the Kuopio University Area Research Ethics Board. Prior to participation, written informed consent was obtained from the patient or the patient’s legally authorized representative if the patient was unable to give consent due to impaired capacity caused by stroke or TIA.

### Study Design and Patients

Between March 2005 and November 2009, our neurologists recruited 162 patients who had been admitted to Kuopio University Hospital with suspected cardioembolic stroke/TIA. Patients whose symptoms were not explained by a hemodynamically significant (>50%) carotid/vertebral artery stenosis, or by AF diagnosed previously (or during hospitalization), were considered for the study. Thirteen patients were excluded due to technical errors or because they refused to participate having previously given informed consent. Altogether 149 patients (47 females; mean age 61 years; range 32–84 years) with suspected cardioembolic stroke/TIA underwent CT-angiography performed with ECG-synchronized mid-diastole cCT (16- or 64-slice, 120 kV, 190 mAs), enabling cardiac volumetric analyses. Transthoracic echocardiography (TTE) and 24-hour Holter ambulatory ECG were performed [12]. The proportions of visceral (VAT), intrathoracic (IAT) and pericardial (PAT) adipose tissue were measured.

### Determinants for LAA Volume

**Figure 1:**
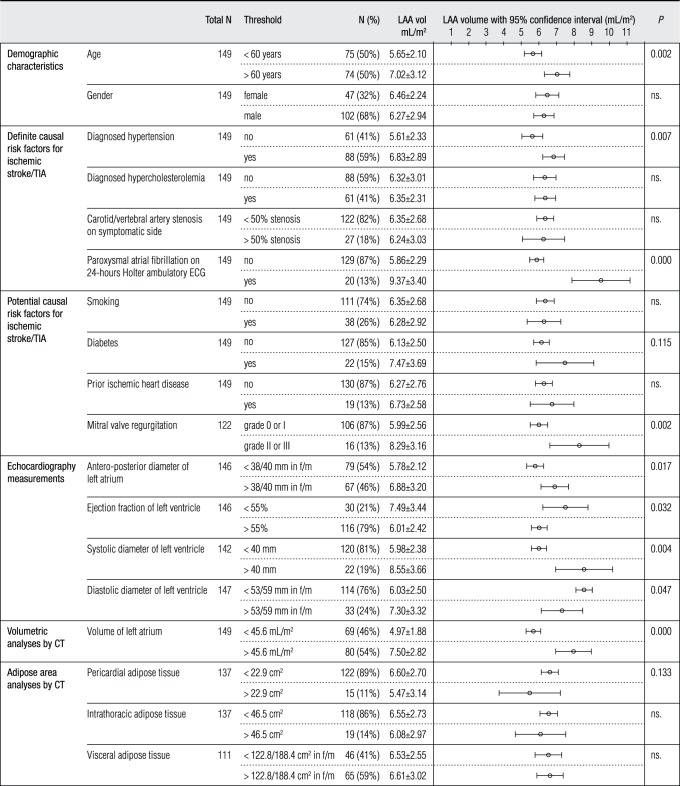
The left atrial appendage (LAA) volume was measured in 149 cardiogenic stroke/TIA patients without chronic atrial fibrillation. There were 18 different variables to determine factors that increase LAA volume. Patients were dichotomized by the measurements performed with transthoracic echocardiography following the guidelines of the American Society of Echocardiography (ASE) [23] and computed tomography (CT) by using the threshold values that were based on normal variations in reference populations [5], [24]. Numbers (N) and percentages (%) of the patients’ mean LAA volumes adjusted for patient’s body surface area with standard deviation in dichotomized groups are presented. LAA volumes with 95% confidence interval in every group are shown. Variables with statistical significance (*P*) <0.2 were included for stepwise linear regression analyses. Vol = volume; f = female; m = male.

Study patients were dichotomized into subgroups according to their general characteristics and many other variables. These included the presence of defined (hypertension [13], dyslipidemia [14], carotid stenosis [15], [16], and PAF [17]) and potential (smoking [18], diabetes [19], prior ischemic heart disease [20], and mitral valve insufficiency [21], [22]) causal risk factors for ischemic stroke, cardiac echoparameters, cCT based volume measurements, and adipose tissue measurements (Figure 1).

For dichotomization of mitral valve insufficiency, grade II mitral regurgitation was used as a threshold. Dimensions in echoparameters and ejection fraction were dichotomized by the guidelines of the American Society of Echocardiography (ASE) [23]. To derive upper thresholds for cCT based measurements, 243 consecutive patients who underwent coronary CT-angiography (coronary CTA) to exclude coronary stenosis were evaluated as candidates for healthy controls. Subjects with coronary stenosis of >50%, AF, hypertension, renal insufficiency, prior stroke or malignancies were excluded, which left 40 patients available to create age- and gender-matched pairs with our stroke/TIA patients [5]. The upper thresholds for normal variances in LAA and LA volume were set to two standard deviations above the mean of the control population. Upper thresholds for IAT and PAT were based on the same control population. The upper thresholds for VAT (>122.8 cm^2^ in females; >188.4 cm^2^ in males) were based on results from an ethnically comparable North American population (n = 1160) [24].

### Volumetric Analysis of the LAA and LA

Quantitative image analysis was performed on an IDS5 diagnostic workstation (version 10.2P4; Sectra Imtec, Linköping, Sweden) by an independent observer (MT), guided by an experienced cardioradiologist (PS). The volumetric analyses of LAA were performed three-dimensionally using the cCT stack. Planimetration of LAA covered 10.4±2.0 consecutive slices in the transversal plane. A two-chamber view and localizer tool were used to differentiate the LAA orifice from the LA. The LAA borders were traced manually on each transverse slice while the LA borders were traced from the consecutive slices on two-chamber plane using the mitral valve annulus as the landmark differentiating the LA from the LV. LAA volume was calculated with Simpson’s method by multiplying each manually traced LAA and LA area by the section thickness (3 mm) and summing up the volumes of the separate sections [25]. Volumes of the LAA are presented with adjustment for BSA, which was calculated using Mosteller’s formula [11]. In our previous study intra-class correlations (ICC) for the LAA volume measurements resulted in almost perfect reproducibility between different observers (ICC = 0.96) and the same observer (ICC = 0.95) [5].

### Measurements of Pericardial, Intrathoracic and Visceral Adipose Tissue

The PAT area was calculated by drawing a line through the parietal layer of the pericardial sac. The IAT area was calculated via the parietal layer of the pleural cavity from sternum to the anterior surface of the ascending aorta. The IAT included all PAT. Both measurements were performed from a single axial slice (slice thickness 7.5 mm) from the opening level of the common left coronary artery. The VAT area (slice thickness 10 mm) was calculated by drawing a line within the muscle wall that delineated the abdominal cavity at the level of the fourth lumbar vertebra. The adipose tissue surfaces were computed with an attenuation range from −30 to −190 Hounsfield Units.

### Statistical Analyses

Continuous variables with normal distribution are presented as mean±SD, and categorical variables are presented as absolute values and percentages. Student’s *T*-test for individual samples was used to compare LAA volumes between dichotomized groups. Inclusion criteria for multifactoral linear regression analyses were set at *P*<0.2, statistical significance at *P*<0.05 and high statistical significance at *P*<0.01. In multivariate analysis, tolerance>0.2 was used to indicate non-multicolinearity of variables. By using the stepwise method, variables were entered if F<0.05 and removed if F>0.1. The change in R^2^ and adjusted R^2^ value were used to assess the contribution of each variable to the LAA volume. Data were analyzed using SPSS for Windows (version 19, 1989–2010 SPSS Inc., Chicago, USA).

## Results

Table 1 shows the clinical characteristics and uncategorized values of echocardiography and CT measurements of the patients. Only BSA-adjusted values were used in regression analyses.

**Table 1:** Clinical Characteristics of Patients with Suspected Cardioembolic Stroke/TIA.

Characteristic	N	Value
Age, yrs	149	60.9±10.6
Females, n (%)	149	47 (31.5)
Body mass index, kg/m^2^	149	28.1±4.4
Body surface area, m^2^	149	2.0±0.2
Caucasian race, n (%)	149	149 (100.0)
Hypertension, n (%)	149	88 (59.1)
Hyperlipidemia, n (%)	149	61 (40.9)
Diabetes, n (%)	149	22 (17.8)
Smokers, n (%)	149	38 (25.5)
Prior myocardial infarction, n (%)	149	19 (12.8)
Prior stroke, n (%)	149	29 (19.5)
No mitral regurgitation in TTE, n (%)	122	59 (48.4)
Grade I mitral regurgitation in TTE, n (%)	122	47 (38.5)
Grade II mitral regurgitation in TTE, n (%)	122	15 (10.1)
Grade III mitral regurgitation in TTE, n (%)	122	1 (0.8)
Left atrium antero-posterior diameter on TTE, mm	146	39.9±6.8
Left ventricle ejection fraction, %	146	62.6±10.7
Left ventricle end-systolic diameter on TTE, mm	142	32.9±7.2
Left ventricle end-diastolic diameter on TTE, mm	142	50.5±6.6
Left atrium volume on CT, mL	149	94.8±32.1
Left atrium volume on CT adjusted for height, mL/m	149	55.3±18.1
Left atrium volume on CT adjusted for BSA, mL/m^2^	149	48.4±16.0
Left atrial appendage volume on CT, mL	149	12.3±5.2
Left atrial appendage volume on CT adjusted for height, mL/m	149	7.2±3.0
Left atrial appendage volume on CT adjusted for BSA, mL/m^2^	149	6.3±2.7
Pericardial adipose tissue area on CT, cm^2^	137	13.5±7.1
Intra-thoracic adipose tissue area on CT, cm^2^	137	29.2±14.1
Visceral adipose tissue area on CT, cm^2^	111	192.3±92.6

Threshold values of LA volume, PAT and IAT area were based on the control population. In comparison between 40 age- and gender-matched stroke/TIA patients and control subjects, no significant difference was found in hyperlipidemia, diabetes, EF, LV dysfunction (no identified wall dyskinesia) or smoking. Body mass index (BMI) was 28.7±4.8 kg/m^2^ in stroke/TIA patients and 25.3±4.1 kg/m^2^ (*P* = 0.002) in control subjects. BSA was 2.0±0.2 m^2^ in stroke/TIA patients and 1.8±0.2 m^2^ (*P* = 0.033) in control subjects. All 18 threshold values are shown in Figure 1.

### Correlates of LAA Volume in Linear Regression Analysis

The possible correlates of LAA volume adjusted for BSA (mL/m^2^) are shown in Figure 1. In univariate analyses, age, previously diagnosed hypertension, atrial fibrillation seen by 24-hour Holter ambulatory ECG, diabetes, mitral valve insufficiency, antero-posterior diameter of the LA, EF, LVSD and LVDD, LA volume and LV PAT were associated with LAA volume (*P*<0.2) and were included in our multivariate linear regression analyses. No significant multi-colinearity (tolerance 0.473–0.892) was observed between the variables.

To investigate independent predictors of LAA volume, stepwise linear regression analysis was performed. The best model accounted for 33% of the variability in LAA volume, whilst AF accounted for 19% (*P*<0.001); enlarged LA volume for 7% (*P* = 0.011); enlarged LVSD for 4% (*P* = 0.007) and decreased PAT for 3% (*P* = 0.043) of the variability. When adjusted for the number of predictors, these variables accounted for 19%, 6%, 3% and 2% of the variability in LAA volume, respectively. The whole model accounted for 30% of the LAA volume variability when adjusted for the number of predictors.

## Discussion

An enlarged LAA constitutes an established risk factor for cardioembolic stroke [7]. In a recent study, over half of all cryptogenic stroke/TIA patients had an enlarged LAA, indicating that cardioembolism may play a role in the mechanism of stroke in these patients [5]. Chronic AF and PAF are generally considered causative factors for secondary LAA enlargement [7]. To the best of our knowledge, no previous study has explained correlates of LAA volume increase in patients with stroke/TIA. We found that previously recognized risk factors for ischemic stroke/TIA combined with routine echoparameters and more novel CT parameters explain together only 30% of LAA volume increase. These factors proved to be AF, enlarged LA volume, enlarged LV systolic diameter and decreased pericardial fat. The prevalence of these risk factors could naturally explain LAA dilatation and furthermore the formation of thrombus in the LAA, which contribute to the pathogenic mechanism of cardioembolic stroke/TIA. In particular, PAF may easily remain unrecognized in routine clinical evaluation [26]. Therefore, in patients with stroke/TIA, assessment of LAA size with cCT might help to identify patients who should be more carefully monitored for possible AF.

Atrial fibrillation explained 19% of the LAA volume variation in the present study. It has been suggested that PAF plays a vital role in LAA volume increase even if not diagnosed with current methods [26]. In our study, AF diagnosis was based on 24-hour Holter ambulatory ECG. It is likely that even more PAFs would have been recognized by using lengthened ECG monitoring such as 7-day Holter ambulatory recording or event recording. The volume increment of the LAA due to AF has been verified by MRI studies. Stroke/TIA patients with PAF in our study (9.37±3.40 mL/m^2^ when adjusted for BSA corresponding to 18.74±6.80 mL when non-adjusted) proved to have even larger LAA volumes compared to patients with PAF (13.0±6.1 mL) or chronic AF (14.3±6.2 mL; 15.04±07.1 mL; 17.3±6.7 mL) in those previously published MRI studies [27], [28], [29].

Second to AF, LA dilatation had an impact on LAA size, explaining 6% of the volume increase. This proportion remains surprisingly small despite the anatomical connection of the structures, indicating that the function and factors influencing their size differ. Indeed, the LAA is not only a component of the LA but it constitutes a separate chamber of the heart with embryological, anatomical, and functional features distinct from those of the LA [30]. On the other hand, LAA enlargement has been shown to be associated with LA filling pressure and hypertension [31]. Being more compliant, the LAA is likely to increase in size prior to LA volume increase [32], [33]. The risk of stroke has been suggested to double for every 10 mm increment in LA diameter in males [34]. Further investigations have confirmed the correlation between stroke/TIA risk and BSA adjusted LA volume enlargement [5].

Our results also show that increased LV systolic diameter on TTE, indicating LV dysfunction, explains 3% of LAA volume increase. In patients with dilated cardiomyopathy, LV dimensions correlate significantly with LAA size [35]. Importantly, in those patients, systolic dysfunction was associated with a high prevalence of LV (13%) and LAA (96%) thrombus in patients with sinus rhythm [35]. These results parallel other studies investigating the relationship between LV systolic function and LAA thrombus formation [36], [37], [38]. We hypothesize that LV dilation not only influences LA dilation and enhances LAA dilation but reduces LAA flow velocity [39]. Elevated filling pressures in the left ventricle contribute to deterioration of the LAA flow, which in turn increases LAA volume [40].

Interestingly, a low amount of pericardial fat was associated with increased LAA volume. The observed impact was minor and our finding is controversial in relation to previously published studies [41], [42]. There appears to be a graded relationship with a higher pericardial fat burden in chronic AF patients compared to PAF patients that is independent of BMI [43]. This may partly explain why a decreased PAT area was found in patients with higher LAA volumes. Patients with chronic AF were excluded from our study while patients with lower volumes of PAT may suffer from PAF [26], [44]. More prospective studies for the evaluation of causalities between LAA volume, arrhythmias and adipose tissue are highly needed. It is to note, that PAT had no statistical significance (*P* = 0.133) in univariate analysis. No conclusions about the role of increased PAT and risk of recurrent stroke can be made from this study.

Our study has some limitations. Firstly, the number of stroke/TIA patients included in the study was relatively small. However, patients were randomly selected without sampling bias. Secondly, 24-hour ECG monitoring is not able to reveal short-term PAFs with long intervals. Therefore, it is likely that PAF plays a more prominent role in cardioembolic stroke/TIA pathogenesis than currently known. Thirdly, the definition of hypertension and dyslipidemia were based on previously established diagnoses, and non-diagnosed cases were not perceived. A diagnosis of hypertension cannot be based on blood pressure measurements in the acute phase of stroke/TIA and comprehensive blood lipoprotein assays were not monitored. Stroke/TIA patients had higher BMI and larger BSA than control subjects. Adjusting the LAA volume with BSA can only decrease the impact of LAA volume increase in stroke/TIA patients compared to controls. This may lead to a negative bias in our hypothesis but does not overstate the result. It has also previously been shown that obesity has no significant impact on these results [5]. While BSA is linked to patients’ weight and hence, to the amount of adipose tissue, adjusting LAA volume with BSA may interfere in the association of PAT area and LAA volume.

To conclude, the extent of LAA enlargement is poorly linked with causative stroke/TIA risk factors and other imaging measurements, implying a relatively independent pathogenic mechanism for cardioembolic stroke/TIA. While PAF recorded with 24-hour Holter ambulatory ECG explains only 19% of LAA increase, it would be ideal to target more extensive ECG monitoring for stroke/TIA patients with large LAA volumes measured by cCT. If a connection between an enlarged LAA and an increased risk for cardioembolic stroke recurrence can be verified in future studies, cCT imaging may help to identify patients for more comprehensive ECG monitoring.
